# Impact of Ce Doping on the Relaxor Behavior and Electrical Properties of Sr_0.4_Ba_0.6_Nb_2_O_6_ Ferroelectric Ceramics

**DOI:** 10.3390/ma18010074

**Published:** 2024-12-27

**Authors:** Yingying Zhao, Pu Mao, Ruirui Kang, Ziao Li, Fang Kang

**Affiliations:** 1 College of Materials Science and Engineering, Xi’an University of Science and Technology, Xi’an 710054, China; liziao2024@163.com; 2 Jiangxi Key Laboratory of Extreme Manufacturing Technology for High-End Equipment, School of Materials Science and Engineering, Nanchang Hangkong University, Nanchang 330603, China; pumao@nchu.edu.cn; 3 Frontier Institute of Science and Technology, State Key Laboratory for Mechanical Behavior of Materials, Future Industrial Innovation Institute of Emerging Information Storage and Smart Sensor, Xi’an Jiaotong University, Xi’an 710049, China; krr0121@xjtu.edu.cn; 4 School of Physics and Electronic Information, Yan’an University, Yan’an 716000, China

**Keywords:** strontium barium niobate, rare earth doping, relaxor behavior, electrical properties

## Abstract

In this work, the rare earth element Ce was incorporated into the A-site of Sr_0.4_Ba_0.6_Nb_2_O_6_ ferroelectric ceramics, which was prepared using the conventional solid state reaction method and sintered under different procedures. A comprehensive investigation was conducted to assess the impact of Ce doping and varying sintering procedures on both the relaxor characteristics and electrical properties of the ceramics. When sintered at 1300 °C for 4 h, the grains exhibited an isometric shape. However, when the sintering temperature increases and the holding time prolongs, the grain size increases and presents columnar crystal. The change tendency of dielectric constant is similar with that of the grain size, and the dielectric peak value of samples sintered at 1300 °C for 4 h is the lowest. But the sintering procedure has almost no influence on the Curie point, which notably decreases as the Ce content rises and is primarily governed by the composition. The diffuseness fitting results and the deviation from the Curie–Weiss law indicate that relaxor characteristics increase with the Ce content increasing. The polarization electric (P-E) loops become slimmer with increasing Ce content, verifying the relaxor behavior variation of samples. As a result, the *P*_max_ and *P*_r_ values decrease and the *P*_max_ − *P*_r_ value increases with increasing Ce content. Notably, the energy storage density and efficiency enhance obviously with higher Ce content, which is attributed to the relaxor behavior. Furthermore, at a Ce content of 4 mol%, the P-E loops and energy storage performance exhibit remarkable frequency and fatigue stability. Therefore, this study offers valuable insights into the investigation of relaxor behavior and the influence of rare earth elements on the properties of tungsten bronze-structured ferroelectrics.

## 1. Introduction

Ferroelectrics have been widely researched due to their excellent dielectric and ferroelectric properties and show great application potential in many aspects such as electronics, capacitors, energy storage, and so on [1,2,3]. And, ferroelectric ceramics are widely studied because of their ease of fabrication [4,5]. As far as we know, the sintering procedures and doping are the two main key factors influencing the structure and properties of ferroelectric ceramics [5,6,7,8,9]. The sintering procedures influence the microstructure directly, in turn influencing the properties. Ion doping is usually used to improve the properties of ferroelectric ceramics. Based on the effects induced by doping on ferroelectric properties, two main types dopant have been approved: acceptor and donor [10,11]. The acceptor and donor elements introduced into A-site and B-site have been widely applied to adjust the properties of perovskite ferroelectrics [12,13]. In order to keep the balance of valance, oxygen vacancy is produced after doping with an acceptor element [13,14,15]. Generally, acceptor doping typically induces aging behavior and results in a pinched polarization–electric field loop, as demonstrated in our previous research [16]. In contrast, donor doping usually leads to the creation of cation vacancies, which can affect phase transitions and induce relaxor behavior [17,18].

As reported, donor doping can influence the microstructure, phase transition, and electrical properties, and induce diffuse transition in perovskite ferroelectrics. For instance, Saburi et al. and Huybrechts et al., introduced La into A-site and Nb into B-site of BaTiO_3_ ceramics, causing these ferroelectric ceramics to transform into semiconductors [19,20]. Hagh et al. doped Sr^2+^ into KNN to replace Na^+^ and K^+^, effectively suppressing the formation of oxygen vacancies and reducing the coercive field [21]. Furthermore, rare earth elements are frequently utilized as donor dopants to enhance the properties of ferroelectrics. Pavlović et al. doped the rare earth elements Ce and La into Bi_4−x_A_x_Ti_3_O_12_, finding that Ce-doped samples exhibited a diffuse transition, while La-doped samples displayed superior dielectric properties [22]. Prasun and his research group introduced rare earth elements into the tungsten bronze-structured Ba_5_RTi_3_Nb_7_O_30_, observing that the dielectric constant increased and the Curie temperature decreased with the dopant radius [23]. Additionally, Li. et al. introduced Bi_2_O_3_ into Sr_0.4_Ba_0.6_Nb_2_O_6_, obtaining a greatly improved dielectric constant, reduced dielectric loss, and slimmer P-E loop [24]. Rare earth element-doped tungsten bronze-structured ferroelectrics often exhibit a diffuse-type of ferroelectric–paraelectric phase transition [25]. Shur and his group obtained nanodomain structures and relaxor behavior in Ce-doped Sr_0.61_Ba_0.39_Nb_2_O_6_ single crystal [26]. It is evident that incorporating donor dopants into tungsten bronze-structured ferroelectrics can induce a diffuse transition, leading to a slimmer P-E loop.

Tetragonal tungsten bronze (TTB)-structured ferroelectrics garner considerable attention, ranking second only to perovskite ferroelectrics [27,28]. Among TTB systems, strontium barium niobate (Sr_x_Ba_1−x_Nb_2_O_6_, SBN) ceramics stand out due to their excellent ferroelectric, dielectric, and pyroelectric properties [29]. The complex TTB structure, featuring tetragonal sites A1, pentagonal sites A2, and triangle sites C, offers many flexibilities for doping. Typically, A1 is usually occupied by Sr ions, while A2 can be occupied by both Sr and Ba ions, and C sites are always empty [30,31]. In our previous work, the SBN transform from normal ferroelectrics to relaxor ferroelectrics with the Sr/Ba ratio increasing [32]. This transition is attributed to the increasing occupation of A2 sites by both Sr and Ba ions, which enhances the disorder degree of A sites [33]. The disorder of A-site ions in SBN has been reported as the primary factor inducing relaxor behavior in these ceramics [34,35]. However, further exploration is necessary to understand the effects of introducing various concentrations of rare earth elements into the A sites of SBN.

Therefore, in this paper, the rare earth element Ce was introduced into A-site of strontium barium niobate system to investigate the effects of donor doping on the electrical properties and relaxor behavior. The Sr_0.4−3x/2_Ba_0.6_Ce_x_Nb_2_O_6_ (x = 1, 2, 4 mol%) ceramics were prepared using conventional state reaction method sintering with different temperatures and holding times. The influences of the sintering procedure and Ce content on the dielectric properties, relaxor behavior, and energy storage performance were investigated. The microstructure of SBN ceramics sintered with different procedures were detected and compared. The temperature-dependent dielectric constant was measured and the corresponding relaxor behavior was analyzed. The ferroelectric measurement verifies the strong relaxor behavior of composition doping with high content Ce. The corresponding temperature-dependent, frequency-dependent, and fatigue resistance properties of the energy storage were also demonstrated. This work can provide some knowledge for the study of effects induced by donor doping on tungsten bronze-structured ferroelectric ceramics.

## 2. Materials and Methods

The conventional solid state reaction method was used to prepare the ferroelectric ceramics Sr_0.4−3x/2_Ba_0.6_Ce_x_Nb_2_O_6_ (abbreviated as SBN40-xCe, x = 1, 2, 4 mol%). The starting materials, BaCO_3_ (99.9%), SrCO_3_ (99.9%), Nb_2_O_5_ (99.9%), and CeO_2_ (99.9%) with high purity, obtained from Alfa Aesar located at Ward Hill (MA, USA), were weighed according to the stoichiometric formula. The weighed powders were mixed and ball-milled thoroughly with ethanol for 4 h. The slurry was put into the oven to dry, then calcined at 1200 °C for 2 h in a muffle furnace. Afterwards, the calcined powders were ball-milled again for another 4 h and dried again. Then, the dried powder was mixed with PVA aqueous solution and pressed into pellets with a 12 mm diameter die. The final sintering temperature was 1300–1350 °C with different holding times of 4–12 h. In the end, the sintered ceramics were coated with Ag electrode to conduct electrical measurements.

A scanning electron microscope (FEI-Q25, FEI Company, Portland, OR, USA) was used to observe the microstructure and Nano Measure software 1.2.0 was used to analyze the grain size. The room temperature phase structure of SBN40-xCe samples was measured using X-ray diffraction (XRD, X’Pert diffractometer with Cu Kα λ-0.15406 nm, Rigaku Corporation, Tokyo, Japan). The XPS (X-ray photoelectron spectroscopy) spectra were acquired using an Axis Ultra instrument from the Kratos Corporation in Manchester, UK, employing a monochromatic Al Ka source operating at 150 W, with a voltage of 15 kV and an energy of 1486 eV. The sintered samples were polished to about 0.8 mm and coated with Ag electrode on both surfaces to measure dielectric and ferroelectric performance. The temperature-dependent dielectric properties were characterized over a temperature range of −150–200 °C using HIOKI LCR Hitester (HIOKI Company, Nagano, Japan). The P-E hysteresis loops were measured by a ferroelectric test system (Precision Premier II, Radiant Company, Redmond, WA, USA). The room temperature P-E loops and temperature-dependent P-E loops over −30–70 °C were measured at 10 kHz. The frequency-dependent P-E loop and the fatigue resistance were measured at room temperature.

## 3. Results

**Figure 1:**
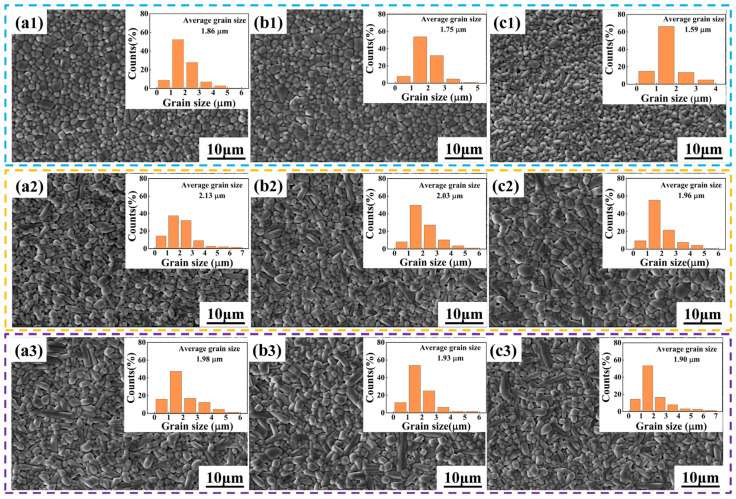
SEM micrographs of SBN40-xCe ceramics sintered under different conditions: (**a1**–**a3**) the SBN40-1Ce ceramics sintered at 1300 °C/4 h, 1300 °C/12 h and 1350 °C/4 h, respectively; (**b1**–**b3**) the SBN40-2Ce ceramics sintered at 1300 °C/4 h, 1300 °C/12 h and 1350 °C/4 h, respectively; (**c1**–**c3**) the SBN40-4Ce ceramics sintered at 1300 °C/4 h, 1300 °C/12 h and 1350 °C/4 h, respectively (Insets show grain size distribution).

To investigate the influences of Ce content and the sintering procedure on the microstructure of SBN ceramics, Figure 1 displays the SEM micrographs of SBN40-xCe ceramics sintered under different conditions. It can be seen clearly that the sintering procedure has an obvious effect on the microstructure of SBN. The grains present isometric crystal when sintering at 1300 °C/4 h. As either the sintering temperature or the holding time is increased, the grain size exhibits a certain degree of increase and the grains present as a columnar crystal. In addition, upon increasing the Ce content, the grain size decreases gradually. This reduction is attributed to the introduction of Ce into the A-site of SBN, which may lead to the formation of cation vacancies located at the grain boundaries. These vacancies inhibit grain growth, ultimately resulting in a decrease in grain size [36].

**Figure 2:**
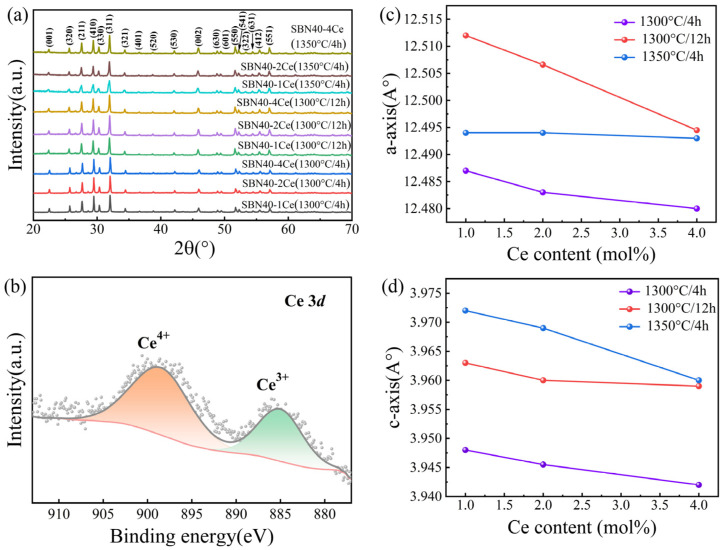
(**a**) XRD patterns of SBN40-xCe ceramics sintered under different conditions, (**b**) Ce 3*d* XPS spectra of SBN40-4Ce sample sintered at 1350 °C/4 h (the gray dots are the measured data and the line is the fitting result). Variation of lattice parameters in (**c**) a-axis and (**d**) c-axis of SBN40-xCe ceramics sintered under different conditions.

In order to analyze the sintering conditions and Ce doping on the phase structure of SBN40 system, the XRD patterns of all the SBN40-xCe samples are shown in Figure 2a. It can be seen that all the samples sintered under different conditions exhibit pure tetragonal tungsten bronze structure without a secondary phase, which verifies the successful incorporation of cerium into the lattice of SBN. Moreover, the Ce 3*d* XPS spectra of SBN40-4Ce sample sintered at 1350 °C/4 h has been investigated to analyze the valence of Ce at high temperature, which is depicted in Figure 2b. One can see that some cerium ions change from the oxidation state 4+ to 3+ while processing at high temperatures. This is in agreement with the results of previous reports that cerium ions tend to be 3+ in SBN systems and substitute for Sr^2+^, occupying the A-site of SBN [37,38]. To determine the influences of Ce doping on the lattice, the calculated lattice parameters in the a-axis and c-axis are exhibited in Figure 2c,d. It can be found that both a and c decrease with the increasing Ce content for all the temperatures, which is in agreement with the references. This is because the Ce^3+^ ions occupy the A-sites and they exhibit a stronger coulomb attraction to the adjacent O^2−^ ions compared to Sr^2+^ and Ba^2+^ [37,38]. In addition, the radius of Ce^3+^ (0.103 nm) is smaller than that of Sr^2+^ (0.113 nm) and Ba^2+^ (0.135 nm), which also makes a contribution to the reduction of lattice parameters. In addition, one can see that with the sintering temperature and the holding time increasing, the lattice parameters show an increased tendency, which is consistent with the report in reference [39].

**Figure 3:**
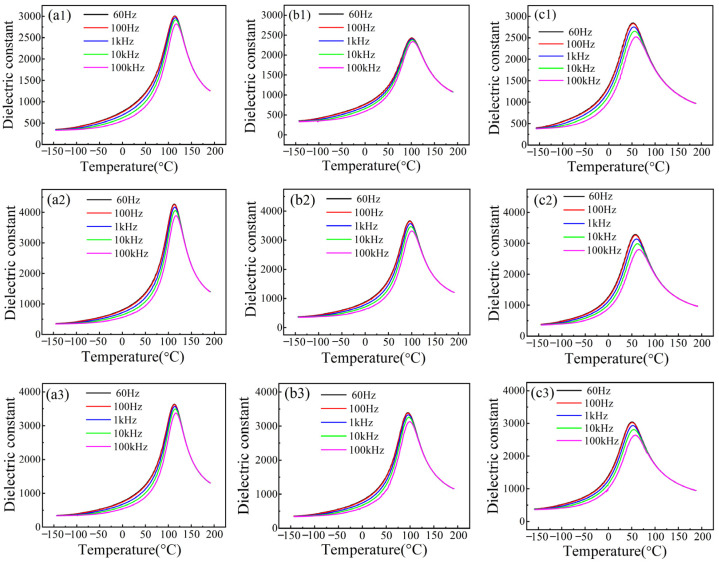
Temperature dependence of dielectric constant of SBN40-xCe ceramics: (**a1**–**a3**) the SBN40-1Ce ceramics sintered at 1300 °C/4 h, 1300 °C/12 h and 1350 °C/4 h, respectively; (**b1**–**b3**) the SBN40-2Ce ceramics sintered at 1300 °C/4 h, 1300 °C/12 h and 1350 °C/4 h, respectively; (**c1**–**c3**) the SBN40-4Ce ceramics sintered at 1300 °C/4 h, 1300 °C/12 h and 1350 °C/4 h, respectively.

The temperature-dependent dielectric constant of SBN40-xCe ceramics sintered under different conditions measured at different frequencies are presented in Figure 3. It is evident that the temperature corresponding to the dielectric peak (*T*_m_) of all the samples shifts towards higher temperature as the frequency increases. Additionally, Figure 3(a1–c1) reveal that as the Ce content increases, the dielectric peak broadens and the frequency dispersion becomes more pronounced for SBN40-xCe ceramics sintered at 1300 °C for 4 h. Similar trends are observed for the samples sintered at 1300 °C for 12 h and 1350 °C for 4 h, as shown in Figure 3(a2–c2,a3–c3). These findings indicate the typical relaxor behavior of SBN40-xCe samples sintered under different conditions. The relaxor response in SBN system has been reported to be closely related with the occupancy and cation movement of the A1 site, which would disturb the A1-O bonds and introduce net displacement in the neighboring Nb polyhedral [40]. In our work, due to the introduction of Ce into the A-site of SBN, the compositional fluctuation increased further compared with pure SBN, which results in a more intensified cation movement. Therefore, the A1-O bonds will be disturbed and the neighboring Nb displacement increases leading to the increased relaxor behavior.

**Figure 4:**
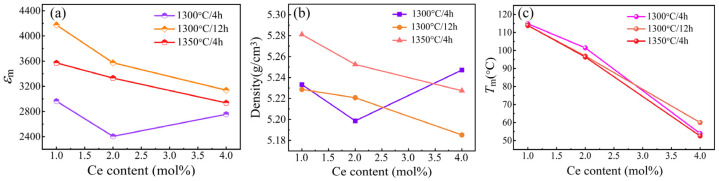
(**a**) Dielectric constant peak value at 1 kHz, (**b**) The density of all the SBN40-xCe samples sintered at different procedures, (**c**) *T*_m_ value at 1 kHz of SBN40-xCe ceramics sintered under different conditions.

Based on the dielectric curves presented in Figure 3, the function of dielectric peak value with the Ce content and the temperature of the maximum dielectric constant (*T*_m_) with the Ce content of SBN40-xCe ceramics sintered under different conditions are depicted in Figure 4a,b, respectively. Generally, it is observed that the dielectric constant peak values of samples sintered at 1300 °C/12 h and 1350 °C/4 h are higher than that of samples sintered at 1300 °C/4 h. This correlation can be attributed to the grain size, as inferred from the grain size distribution results shown in Figure 1. When the sintering procedure is 1300 °C/12 h and 1350 °C/4 h, the variation of dielectric peak value is same as the change tendency with the grain size. This is also caused by the grain size change. For the samples sintered at 1300 °C/4 h, the dielectric constant decreases first and then increases. This may be because the porosity of the SBN40-2Ce sample influences the dielectric constant [7]. Moreover, the density of all the SBN40-xCe samples sintered under different conditions are shown in Figure 4b. It is evident that the change tendency of the density with Ce content is similar with the dielectric constant, which is a contributing factor to the low dielectric constant of the SBN40-2Ce sample at 1300 °C/4 h. For the change tendency of *T*_m_ with Ce content shown in Figure 4c, the *T*_m_ shows a little difference for samples sintered under different conditions, while the value decreases obviously with increasing Ce content. These results state that the sintering procedure has almost no influence on the transition temperature *T*_m_, which mainly depends on the composition.

Equations (1) and (2) listed as follows are usually used to describe and analyze the dielectric characteristic of normal ferroelectrics and relaxor ferroelectrics [41,42,43,44]:

(1) $$1/\varepsilon \;=\;(T\;-T_{0})/C,\;(T\;>\;T_{C})$$

(2) $$\Delta T\;={\;T}_{B}-T_{m}$$

where *ε* represents the dielectric permittivity at the temperature of *T*, *T*_0_ is the Curie temperature, and C describes the Curie constant; *T*_B_ denotes the temperature of the dielectric constant starts deviate from the Curie–Weiss law, *T*_m_ is the temperature corresponding to the maximum dielectric constant, and ∆T describes the deviation degree of dielectric constant. As far as we know, the dielectric constant of normal ferroelectrics obeys the Curie–Weiss law, as shown in Equation (1) over the temperature range higher than Curie point. However, the dielectric constant of relaxor ferroelectrics always deviates from the Curie–Weiss law because of the presence of PNRs. Above the Burns temperature (*T*_B_), which is higher than *T*_m_, the dielectric constant of relaxor ferroelectrics obeys the Curie–Weiss law. Conversely, below *T*_B_, the dielectric constant deviates from the Curie–Weiss law [45,46]. The degree of this deviation is often quantified using ∆T, as defined in Formula (2). However, the deviation is related to the dislocations, grain interfaces, and other structural defects, which are strongly temperature dependent. This leads to the parameters obtained depending on fitting temperature range, which was actually different in different works [42,47]. The deviation from the Curie–Weiss law of SBN40-xCe ceramics sintered at 1350 °C for 4 h are demonstrated in Figure S1a–c. It can be seen that the ∆T value increases with increasing Ce content, indicating the boosting relaxor behavior of SBN40-xCe ceramics.

In addition, the modified Curie–Weiss law shown in Equation (3) can also be used to demonstrate the diffuseness of ferroelectrics [48].

(1/*ε* − 1/*ε*_m_) = (*T* − *T*_m_)*^γ^*/*C* (3)

where *ε*_m_ is the peak value of the dielectric permittivity at the temperature *T*_m_ and the *γ* (1 ≤ *γ* ≤ 2) value exhibits the diffuseness of ferroelectrics. Usually, the normal ferroelectrics obey Curie–Weiss behavior and the diffuseness value *γ* is 1, while *γ* = 2 usually represents ideal relaxor ferroelectric. It can be found from Figure S1d–f that the fitting is very good and the diffuseness values *γ* of all the samples are all close to 2, which are higher than that of the pure SBN40 reported in reference [49,50]. This verifies the relaxor behavior induced by the introducing of rare earth Ce into SBN ceramics. However, given that the *γ* value can be affected by extrinsic factors, including composition homogeneity and the range of fitting temperatures, it serves as an imperfect and occasionally unreliable measure of relaxor behavior [41]. Consequently, we have introduced an additional parameter, ∆*T*_relaxor_, to quantify the degree of relaxor behavior more accurately. The ∆*T*_relaxor_ can be calculated based on the Equation (4), as detailed in references [41,51], and the results are presented in Table 1.

(4) $$\Delta T_{\mathrm{relaxor}}=T_{\varepsilon_{m}\left(100\mathrm{kHz}\right)}-T_{\varepsilon_{m}\left(100\mathrm{Hz}\right)}$$

The results indicate that as x increases, the ∆*T*_relaxor_ value increases, implying a progressive augmentation in the degree of relaxor behavior. This finding underscores the pronounced relaxor characteristics displayed by the SBN40-4Ce sample. Additionally, Table 1 presents both the deviation value and the diffuseness fitting results. The increased relaxor behavior of SBN40-xCe ceramics is considered to be related to the increased ionic disorder degree of the A-site, resulting from the introduction of Ce.

**Figure 5:**
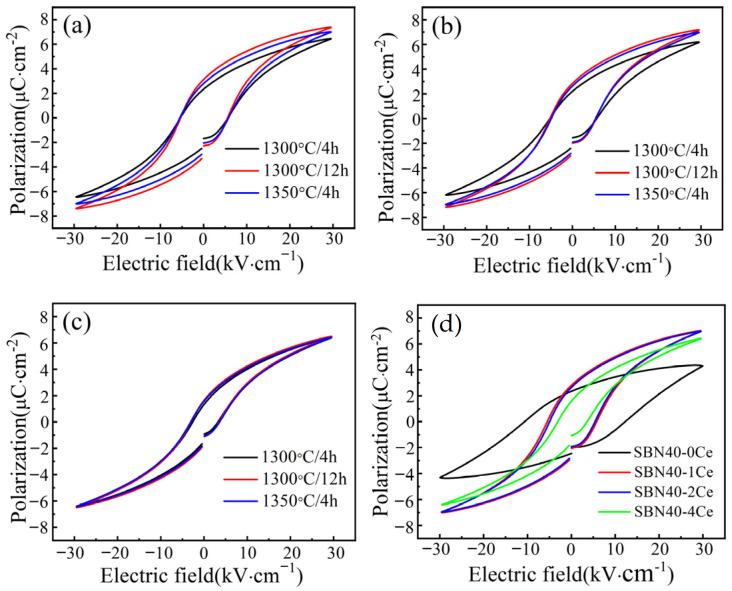
P-E loops of SBN40-xCe ceramics sintered under different conditions: (**a**) SBN40-1Ce, (**b**) SBN40-2Ce, (**c**) SBN40-4Ce, (**d**) Comparison of P-E loops of SBN40-xCe ceramics at 1350 °C/4 h.

In order to explore the effects of introducing Ce on the ferroelectric properties and energy storage performance, the P-E loops of SBN40-xCe samples sintered under different conditions measured at 10 Hz are shown in Figure 5a–c. It can be found that the sintering procedure has little influence on the P-E loop. For the SBN40-1Ce and SBN40-2Ce compositions, the maximum polarization value *P*_max_ of the samples sintered at 1300 °C/12 h and 1350 °C/4 h are higher. For the SBN40-4Ce, the P-E loops of different sintering procedures remain almost unchanged regardless of the sintering procedure. For comparison, the P-E loop of the SBN40-0Ce sample and the P-E loops of SBN40-1Ce, SBN40-2Ce, and SBN40-4Ce samples sintered at 1350 °C/4 h are shown in Figure 5d. Notably, all Ce-doped samples demonstrate higher *P*_max_ values compared to the SBN40-0Ce sample, suggesting that Ce doping enhances the polarization of SBN system. Additionally, the Ce-doped samples exhibit slimmer P-E loops compared to the undoped sample, indicative of the relaxor characteristics induced by Ce doping. Moreover, the P-E loop of the SBN40-4Ce sample shows a much slimmer P-E loop than other compositions, which indicates its strong relaxor behavior. These findings align with the previous analysis of the dielectric response, as well as the ∆T, ${\Delta T}_{\mathrm{relxor}}$, and *γ* value.

**Figure 6:**
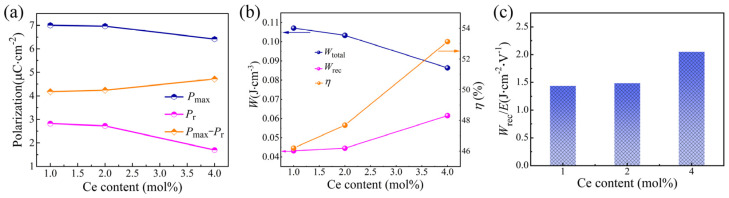
(**a**) Function of *P*_max_, *P*_r_, and *P*_max_ − *P*_r_ with Ce content, (**b**) energy storage performance of SBN40-xCe ceramics, (**c**) comparison of *W*_rec_/*E* of SBN40-xCe ceramics.

In order to analyze the energy storage properties of SBN40-xCe samples, the variations of *P*_max_, *P*_r_, and *P*_max_ − *P*_r_ with Ce content and the energy storage performance of SBN40-xCe samples sintered at 1350 °C/4 h deduced from the P-E loops are investigated. The function of *P*_max_, *P*_r_, and *P*_max_ − *P*_r_ with Ce content are shown in Figure 6a. The results indicate that both *P*_max_ and *P*_r_ decrease as Ce content increases, with *P*_r_ decreasing more significantly than *P*_max_. As a result, *P*_max_ − *P*_r_ exhibits an upward trend as Ce content rises. Based on the P-E loop measurement results, the energy storage density and efficiency are calculated according to the Equations (5)–(7) [52,53].

(5) $$W_{\mathrm{total}}=\int_{0}^{P_{\max}}EdP$$

(6) $$W_{\mathrm{rec}}=\int_{P_{r}}^{P_{\max}}EdP$$

(7) $$\eta =W_{\mathrm{rec}}/W_{\mathrm{total}}$$

in which *W*_total_ is the charge energy stor age density, *W*_rec_ is the recoverable energy storage density and *η* describes the energy storage efficiency. The change tendency of the calculated *W*_total_, *W*_rec_, and *η* results are depicted in Figure 6b. The *W*_total_ decreases with increasing Ce content; however, as the Ce content increases, both *W*_rec_, and *η* exhibit an upward trend, which aligns with the changing tendency of *P*_max_ − *P*_r_, as can be deduced from Equation (6). The composition of x = 4 mol% achieves the highest values for *P*_max_ − *P*_r_, *W*_rec_ and *η*, which is benefited from the strong relaxor behavior exhibited by the SBN40-4Ce sample. To further evaluate the energy storage density of SBN40-xCe samples, we introduce the unit-electric field energy storage density (*W*_rec_/*E*) as a metric and present it in Figure 6c. The trend in *W*_rec_/*E* suggests that Ce doping enhances the energy storage potential of the samples, and all SBN40-xCe samples demonstrate a considerable energy storage capacity.

**Figure 7:**
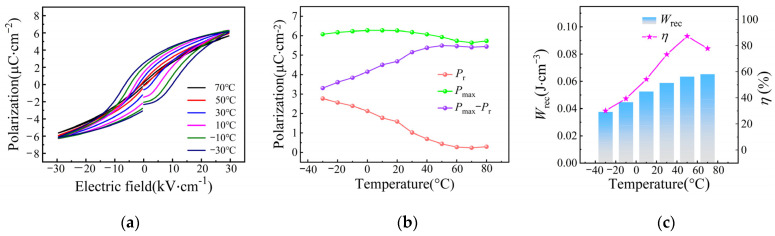
(**a**) P-E loops, (**b**) variation of *P*_max_, *P*_r_, and *P*_max_ − *P*_r_, and (**c**) *W*_rec_ and *η* of SBN40-4Ce samples at temperatures −30–70 °C at 30 kV/cm.

In order to investigate the temperature dependence of ferroelectric and energy storage performance, the P-E loop of the SBN40-4Ce sample measured over the temperature range −30–70 °C is depicted in Figure 7a. The results show that with the measured temperature increasing, the P-E loop becomes slimmer and slimmer. This is because with the temperature increasing, the ferroelectricity is weakened. The temperature dependence of *P*_max_, *P*_r_, and *P*_max_ − *P*_r_ is shown in Figure 7b. With the temperature decreasing, *P*_max_ increases slightly first, then decreases a little. It can be ascribed to the fact that with the temperature decreasing, the SBN40-4Ce sample will transform from the paraelectric phase to the ferroelectric phase, along with *P*_max_ increasing. As the temperature decreases further, the domain switching becomes difficult, so *P*_max_ decreases. *P*_r_ increases with the temperature decreasing, which can be attributed to the SBN40-4Ce sample transforming from paraelectric phase to ferroelectric phase. As a result, *P*_max_ − *P*_r_ decreases obviously with the temperature decreasing. The energy storage performance calculated over the temperature range −30–70 °C is shown in Figure 7c. It can be seen that the energy storage density *W*_rec_ increases with the temperature increasing, and shows a relative stable variation at higher temperature and the same tendency with *P*_max_ − *P*_r_. The energy storage efficiency *η* represents an increasing tendency with the temperature increasing. At higher temperatures, *η* shows a little decrease, which is because *P*_max_ and *P*_r_ both decrease at higher temperatures. In general, the energy storage performance is relative stable at higher temperatures.

**Figure 8:**
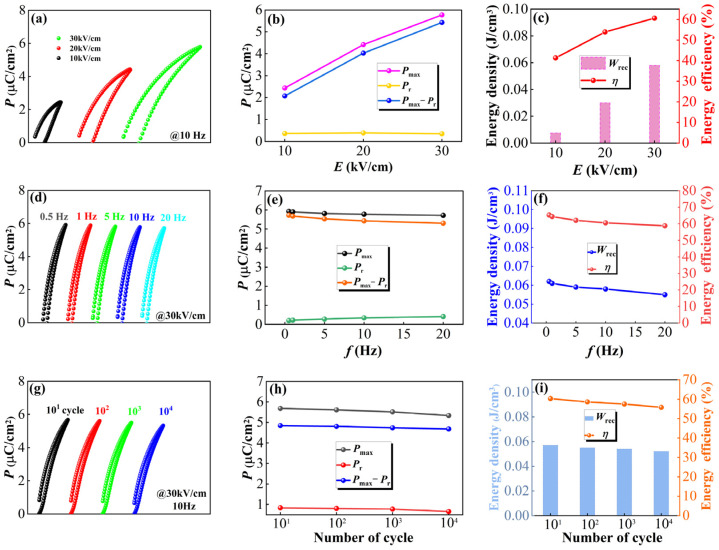
(**a**,**d**,**g**) Unipolar P-E loops of SBN40-4Ce sample sintered at 1350 °C/4 h at different electric fields, frequencies, and cycle numbers. Corresponding *P*_max_, *P*_r_, *P*_max_ − *P*_r_, *W*_rec_, and *η* (**b**,**c**) under various electric fields; (**e**,**f**) under various frequencies; and (**h**,**i**) under different cycle numbers.

To further investigate the energy storage characteristic of SBN40-xCe samples, the unipolar P-E loops of the SBN40-4Ce sample sintered at 1350 °C/4 h under different electrical fields are shown in Figure 8a, and the corresponding variation of *P*_max_, *P*_r_, *P*_max_ − *P*_r_, *W*_rec_, and *η* are depicted in Figure 8b,c. It can be seen that the P-E loop shape changes slightly with the electric field increasing, indicating the stable relaxor behavior of the SBN40-4Ce sample under a different electrical field. As the electric field increases, the *P*_r_ changes a little due to the good relaxor behavior of the SBN40-4Ce sample, and the *P*_max_ and *P*_max_ − *P*_r_ increase due to the PNRs interacting with each other, resulting in an enhanced *W*_rec_ and *η*. In practical applications, dielectric ceramic capacitors frequently operate in a wide range of scenarios, including various working frequencies and extended periods of use. Therefore, the frequency and fatigue stability of the above energy storage parameters for SBN40-4Ce sample sintered at 1350 °C/4 h are also determined and illustrated in Figure 8d–i. Figure 8d–f depicts the unipolar P-E loops measured at frequencies (0.5 Hz–20 Hz) and corresponding *P*_max_, *P*_r_, *P*_max_ − *P*_r_, *W*_rec_, and *η* at 30 kV/cm and room temperature. One can see that the P-E loops keep a slim shape at different frequency and the *P*_max_ and *P*_max_ − *P*_r_ show a slight decrease, as well as the *P*_r_ showing a slight increase. As a result, although *W*_rec_ and *η* show a little decrease with the changing of frequency, the samples show a good frequency stability. The fatigue behavior was evaluated at room temperature, using a frequency of 10 Hz and a voltage of 30 kV/cm, with a cycling number of 10–10^4^. Figure 8g illustrates the P-E loops at different cycles, while Figure 8h,i displays the corresponding variations of *P*_max_, *P*_r_, *P*_max_ − *P*_r_, *W*_rec_, and *η*. It can be found that the P-E loops keep slim after multiple cycles and the values of *P*_max_, *P*_r_, and *P*_max_ − *P*_r_ exhibit minimal variation with increasing cycle number. Consequently, the energy storage parameters *W*_rec_ and *η* undergo a slight decrease as the cycle number increases, indicating the excellent fatigue stability of the SBN40-4Ce sample.

## 4. Conclusions

In conclusion, strontium barium niobate ceramics doped with rare earth element cerium were synthesized by the state reaction method and sintered under different temperatures and times. At a sintering temperature of 1300 °C for 4 h, the grain presents as an isometric crystal. When the sintering temperature or the holding time increasing, the grains show as a columnar crystal. Both the temperature-dependent dielectric constant curves and the diffuseness fitting results demonstrate that the typical relaxor behavior was achieved in the SBN40-xCe ceramics, with the composition x = 4 mol% demonstrating the strongest relaxor characteristics. The change tendency of dielectric constant value is the same as the grain size. Furthermore, the transition temperature *T*_m_ was primarily determined by the composition and showed little correlation with the sintering process. In addition, the SBN40-4Ce sample presents a much slimmer P-E loop than other compositions, indicating its strong relaxor behavior. Consequently, the energy storage performance of SBN40-4Ce sample demonstrate the highest value of *W*_rec_ and *η* at room temperature, along with excellent frequency and fatigue stability. Our work offers invaluable insights for initiating relaxor phase transitions in tungsten bronze structure ferroelectrics by incorporating rare earth elements. Furthermore, it establishes a solid foundation for tailoring the grain morphology of tungsten bronze structures through both doping and adjusting sintering conditions, ultimately leading to an enhancement in their energy storage capabilities.

## Figures and Tables

**Table 1:** Dielectric and relaxor parameters of SBN40-xCe samples of 1350 °C/4 h.

Composition	*T*_m_/K	*T*_B_/K	∆*T*/K	${\Delta T}_{\mathrm{relaxor}}$/K	γ
SBN40-1Ce	387	402	15	3.36	1.80
SBN40-2Ce	370	389	19	4.25	1.89
SBN40-4Ce	326	348	22	7.22	1.71

## Data Availability

Data is contained within the article.

## Supplementary Materials

The following supporting information can be downloaded at: https://www.mdpi.com/article/10.3390/ma18010074/s1, Figure S1: The function of reciprocal of the dielectric constant (at 1 kHz) with temperature (a) SBN40-1Ce (b) SBN40-2Ce (c) SBN40-4Ce; Plots of ln(1/*ε* − 1/*ε*_m_) versus ln(*T* − *T*_m_) at 1 kHz of (d) SBN40-1Ce (e) SBN40-2Ce (f) SBN40-4Ce. (sintering procedure: 1350 °C/4 h).
