# Supplementary material for: Impact of Ce Doping on the Relaxor Behavior and Electrical Properties of Sr0.4Ba0.6Nb2O6 Ferroelectric Ceramics
Source: Materials (Basel). 2024 Dec 27;18(1):74. doi: 10.3390/ma18010074 (PMC11722388; doi:10.3390/ma18010074)
Supplement: Supplementary file 1 [file materials-18-00074-s001.zip › materials-3287695-supplementary.pdf]

Impact of Ce Doping on the Relaxor Behavior and Electrical Properties of $Sr_{0.4}Ba_{0.6}Nb_2O_6$ Ferroelectric Ceramics

Yingying Zhao [1,*], Pu Mao [2], Ruirui Kang [3], Ziao Li [1] and Fang Kang [4,*]

[1] College of Materials Science and Engineering, Xi'an University of Science and Technology, Xi'an 710054, China
[2] Jiangxi Key Laboratory of Extreme Manufacturing Technology for High-End Equipment, School of Materials Science and Engineering, Nanchang Hangkong University, Nanchang 330603, China
[3] Frontier Institute of Science and Technology, State Key Laboratory for Mechanical Behavior of Materials, Future Industrial Innovation Institute of Emerging Information Storage and Smart Sensor, Xi'an Jiaotong University, Xi'an 710049, China
[4] School of Physics and Electronic Information, Yan'an University, Yan'an 716000, China
* Correspondence: yyzhao@xust.edu.cn (Y.Z.); 825384361@yau.edu.cn (F.K.)

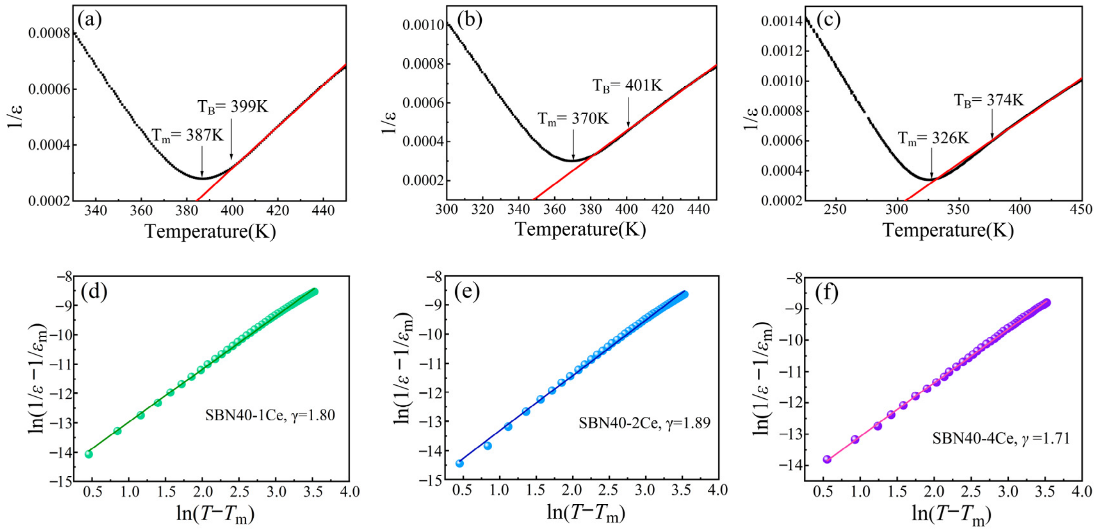

Figure S1. The function of reciprocal of the dielectric constant (at 1 kHz) with temperature (**a**) SBN40-1Ce (**b**) SBN40-2Ce (**c**) SBN40-4Ce; Plots of ln(1/ε-1/ε_m) versus ln(T-T_m) at 1 kHz of (**d**) SBN40-1Ce (**e**) SBN40-2Ce (**f**) SBN40-4Ce. (sintering procedure: 1350 °C/4 h).
